# The Natsal-SF: a validated measure of sexual function for use in community surveys

**DOI:** 10.1007/s10654-012-9697-3

**Published:** 2012-06-19

**Authors:** Kirstin R. Mitchell, George B. Ploubidis, Jessica Datta, Kaye Wellings

**Affiliations:** 1 C/o Rachael Parker, Department of Social and Environmental Health Research, London School of Hygiene and Tropical Medicine, 15-17 Tavistock Place, London, WC1H 9SH UK; 2 Department of Population Studies, London School of Hygiene and Tropical Medicine, Keppel Street, London, WC1E 7HT UK

**Keywords:** Ageing, Community surveys, Measurement, Prevalence, Outcome measure, Sexual function, Sexual dysfunction, Validation

## Abstract

Sexual dysfunction often features as an outcome variable in community health surveys and epidemiological surveys. Key design imperatives for measures included in large scale, population-based surveys are acceptability, brevity and relevance to diverse sexual lifestyles. None of the available measures of sexual dysfunction are entirely suited to this task. We developed a new measure of sexual function for the third British National Survey of Sexual Attitudes and Lifestyles (Natsal 3). Items for the measure were derived from qualitative work from patients and community members. The draft measure was developed and validated using a general population sample (internet panel survey (n = 1,262)) and a clinical sample (patients attending sexual problems clinics (n = 100). Confirmatory factor analysis established that a ‘general-specific model’ had the best fit and was equivalent between general population and clinical samples (Comparative Fit Index = 0.963 Tucker Lewis Index = 0.951; Root Mean Square Error of Approximation = 0.064). The 17-item Natsal-SF is positively associated with the Female Sexual Function Index-6 (B = 0.572) and Brief Sexual Function Questionnaire for men (B = 0.705); it can discriminate between clinical and general population groups (OR = 2.667); and it has good test–retest reliability (r = 0.72). The Natsal-SF provides an estimate of the level of sexual function in the last year. By including items on distress about sex and sexual relationships, and by being relevant to all regardless of sexual lifestyle, it addresses some of the gaps in current measurement design.

## Introduction

Sexual dysfunction often features as an outcome variable in large-scale community health studies and epidemiological surveys of common conditions such as cancer, diabetes and cardiovascular disease. It may also be included as an explanatory variable, for instance in surveys measuring quality of life. Although many measures of sexual dysfunction exist [1–3] there is neither a standard measure nor obvious choice of measure for inclusion in such surveys.

In the context of a community survey, the design imperatives for a measure of sexual dysfunction are demanding. They include acceptability, brevity [4], and relevance to diverse sexual lifestyles. Of the currently available measures, none has been specifically designed to measure prevalence in the community. Perhaps because of this, none is entirely suited to the task [5]. Previously we identified and assessed 54 psychometric measures and did not find a suitable measure with equivalent male and female versions (Mitchell, unpublished thesis). As we have suggested elsewhere [5], the most widely used male and female measures each have limitations with respect to community surveys. The Female Sexual Function Index (FSFI) [6] is perhaps the most widely known among validated measures for women. Although fairly brief (19 items), it asks only about function in the past four weeks and does not ask about the degree of distress related to symptoms. The International Index of Erectile Function (IIEF) [7] comes close to a gold standard for men. Again it is brief (11 items) but it is focused on erectile function, could be viewed by some as intrusive (e.g. ‘how often were your erections hard enough for penetration?’), is less relevant to gay men (because several items assume vaginal penetration), and also does not measure the degree of distress related to symptoms.

This paper describes the development and validation of a new measure designed to assess the prevalence of sexual function problems in the community. In designing the measure we were guided by the definition of sexual dysfunction formulated by the World Health Organisation (WHO): “The various ways in which an individual is unable to participate in a sexual relationship as he or she would wish. Sexual response is a psychosomatic process and both psychological and somatic processes are usually involved.” [8, p 191]. It is generally not feasible nor desirable for community surveys to measure clinical dysfunction as this requires a clinical diagnosis (including detailed information on aetiology in order to rule out organic causes) [9, 10]. Community based studies that measure sexual problems but report them as sexual dysfunction, have met with criticism [11]. We focused instead on sexual function, which we defined as the inverse of the WHO definition of dysfunction: the extent to which an individual is able to participate in a sexual relationship as he or she would wish. We contend that sexual function is about more than just the absence of sexual function problems. Our previous development work for this study [5], suggests that it is also about a positive and healthy sexual relationship, as well as enjoyment, sexual satisfaction and an absence of distress.

Our desire to develop a measure of sexual function for use in community surveys was prompted by our work on the third British National Survey of Sexual Attitudes and Lifestyles (Natsal 3). This is a large, ten yearly, national stratified probability sample survey, and one of the largest face-to-face surveys of sexual behaviour in the world [12–14].

## Methods

### Conceptual framework and item selection

The content of the measure was designed with significant input from patients and community members. We sought to design a conceptual framework for the measure based on their views and experiences. We conducted 32 semi-structured interviews with community members as well as with individuals who had sought help for sexual function problems. Maximum variation sampling was used to ensure a wide range in terms of experience of sexual difficulties. Individuals were recruited from: a sexual problems clinic (n = 6; clinical sample); the diabetes and depression patient lists of a General Practice (n = 13; community members at higher risk of difficulties); an HIV charity (n = 3; community members at higher risk of difficulties); and the waiting room of a General Practice (n = 10; community sample). As is usual for qualitative methodology, the sample size was small to allow in-depth exploration of the data. The interviews explored the range of criteria used by participants in assessing their sex lives and what was seen, and not seen, as problematic. Interview transcripts were coded to identify potential criteria for a functional sex life. Based on the qualitative data and academic literature, and following a set of decision rules, extraneous criteria were excluded. The rules were:

1. If two criteria overlap, exclude the criterion for which the evidence is weakest.
2. Exclude any criterion that interview respondents regarded as desirable rather than essential.
3. Exclude criteria that are associated with sexual function, rather than part of the construct itself.

The second rule, stipulating a focus on the essential, reflected our design imperatives of brevity and public health utility [5]. The last rule involved differentiating correlates of sexual function from the criteria representing the construct itself. We defined as correlates any criteria that could be construed as antecedent to, or an outcome of, a functioning sex life or criteria that were “a degree or so removed from explicit sexual behaviour” [15, p 293]. The methodology for this qualitative stage of the study is described in detail elsewhere [5].

The measure was designed as a computer-based instrument (for completion by respondent or interviewer). The rationale for this was threefold: firstly, the measure is primarily designed for use in Natsal 3, which is a computerized survey; secondly, in future the measure is most likely to be used in large-scale health surveys, which increasingly use computers; and thirdly, a computer-based design allowed more complex filtering, providing the flexibility to cater for wide variation in individual sexual experience. The selected criteria were translated into draft items. Some items (Q9 and several items under Q1) were similar to items in the previous Natsal survey but the others were newly created, following a review of items in existing measures. The items were pre-tested to investigate: acceptability; comprehension, correspondence between respondents’ actual experience (as reported in interview) and their questionnaire responses; and efficiency of routing and question order.

At the piloting stage, 12 interviews were conducted with individuals sampled from a general practice waiting room (a proxy for the general population); and four were conducted with individuals from a sexual problems clinic (clinical sample), both situated in North London. After completing the measure, participants reviewed their answers with an interviewer. Cognitive techniques (for example, thinking aloud; rephrasing in the respondents own words) were used to elicit participant views on the measure. The methodology and results of the pre-test are described in further detail elsewhere (Mitchell and Datta, unpublished study report).

### Measure formation and validation

We implemented a survey to test the draft items and select those with the strongest psychometric properties for inclusion in the final measure; and to test the reliability and validity of the final measure.

### Sample

The survey involved a general population sample (n = 1,262) and a clinical sample (n = 100).

The general population sample was obtained via an internet panel administered by one of the UK’s leading market research companies. The panel has 420,000 or so members living in Britain who collect reward points for participation. Data quality is maintained by validating new members, and by close monitoring of ‘survey behaviour’ to eliminate panellists who give inconsistent responses or who display low engagement (for example, completing surveys too quickly). Panellists for this study were selected randomly within nationally representative quotas on age, gender and region. The survey link was sent to 13,489 members aged 18–74 and data from the first 1,262 completed surveys to fill the quotas were analysed. Of these respondents, 144 completed the measure again 2 weeks later (in order to assess test–retest reliability).

The clinical sample (n = 100) was recruited via four NHS sexual problems clinics in London. Following their consultation, new clinic patients were introduced to the study by their clinician, who gave them an invitation letter and an information sheet with instructions on how to access the web-based survey The majority of patients completed the survey at home after their clinic appointment. In one clinic some respondents opted to complete the survey on a computer in a private room in the hospital. Respondents were given £10 worth of shop vouchers, as thanks for their contribution to the study.

### Comparison measures and variables

The online questionnaire included all the items from our new measure, plus several items for comparison (variables that in theory should correlate with sexual function (see Table 1). We also included two existing measures of sexual function.

**Table 1:** Variables examined for association with the Natsal-SF

Variable	Response options
*Health status*	
General health	1. Excellent 2. Good 3. Fair 4. Bad
Health condition or disability affecting sexual activity or enjoyment	1. Yes 2. No
Use of medication limiting sexual activity or enjoyment	1. Yes 2. No
*Frequency of sex*	
On how many occasions in the last 4 weeks have you had sex?	Number typed in
From the BSFQ (men only): In the past 3 months, has the frequency of your sexual activity with a partner been:	1. Less than you desire 2. As much as you desire 3. More than you desire
*Communication about sex*	
Ease of communication about sex with partner	1. Easy with a husband, wife or regular partner, but difficult with a new partner 2. Easy with a new partner, but difficult with a husband, wife or regular partner 3. Easy with any partner 4. Difficult with any partner 5. Depends/Would vary/Can’t say/Don’t know
*Views about sex*	
Importance of a happy sexual relationship to successful marriage or long term relationship	1. Very important 2. Quite important 3. Not very important 4. Not at all important 5. Don’t know
Level of enjoyment of sex	1. I always enjoy it 2. I enjoy it most of the time 3. I don’t often enjoy it 4. I never enjoy it
*Depression*	
Frequency of feeling down, depressed or hopeless in the last two weeks	1. Not at all 2. Several days 3. More than half of days 4. Nearly every day (Recoded to a binary variable, daily or not)
Extent of agreement with statement “Generally speaking I am satisfied with my life at the moment”	1. Strongly agree 2. Slightly agree 3. Neither agree nor disagree 4. Slightly disagree 5. Disagree strongly
Currently use of prescription medicine for depression	Yes/no
*Alcohol use*	
Frequency of alcohol use in last year	1. Five or more days a week 2. Three or four days a week 3. Once or twice a week 4. Once or twice a month 5. Once or twice in the last year 6. Not at all in the last year (Recoded to a binary variable discriminating between frequent (at least 3 times a week) and less frequent use)

As outlined above, there are no universally agreed standard instruments for measuring sexual function in the community. From the array of reliable and valid measures we chose, for comparison, two whose dimensions looked fairly similar to our own. The female comparison measure, the Female Sexual Function Index (FSFI), is well known and has been used extensively [6]. We used the FSFI-6, a validated item-reduced version of this measure [16], in order to minimise questionnaire length and respondent burden. The chosen male comparison measure, the Brief Sexual Function Questionnaire (BSFQ) for men [17] has an emphasis on psychological aetiologies and probes the relational aspect of sexual function without assuming that the respondent has a sexual partner.

Both of the selected measures (the FSFI-6 and BSFQ) ask about sexual function in the last month. In order to provide a fairer comparison with our measure (in which the reporting period is the past year), we extended the reporting period for each measure to the last 3 months; a compromise between comparability and staying close to the original timeframes of the FSFI-6 and BSFQ. We modified the 21 item BSFQ to reduce respondent burden, omitting 9 items. The omitted items were those asked elsewhere in the questionnaire (e.g. frequency of sexual activity), items deemed unessential for comparison purposes (e.g. sexual orientation) and items providing detail not required for comparison purposes (e.g. length of intercourse after insertion of penis and before ejaculation).

### Statistical analysis

Our latent variable measurement models were based on a multivariate probit analysis with latent variables [18] through a 2-parameter normal ogive item response model and its extension to polytomous/ordinal data [19]. In such models, the factor loading reflects the strength of the association between the observed item and the latent construct. The threshold parameter reflects the point of the latent construct that needs to be reached for a particular response option to be endorsed. Within this measurement modelling framework it is possible to estimate an individual’s scores on the Natsal-SF against their standard error of measurement. This plot is a scale information function (SIF) or scale characteristics curve (SCC). The SIF indicates the range of estimated scores for which an item, item response, or scale is most precise for measuring a persons’ level of, in this instance, sexual functioning. The information is Fisher information i.e. statistical information, and relates to the reciprocal of the square root of the posterior standard deviation of the estimated score (posterior mean). It is the same information that is used to construct a confidence interval for an estimated score, under the assumption of a normal distribution underpinning scores. From a SIF we can identify where the standard error is of constant width, and at what point on the measurement continuum standard errors start to increase, indicating less precise measurement. Psychometric results such as these enable a more informed statement to be made about the measurement range of an instrument when applied in a population. For example, it enables the researcher to define the centile range over which estimated scores have a sufficiently small standard error (precision) to be considered a reliable score.

In the second stage of the analysis, the selected measurement model was combined with a set of observed covariates as well as external validation criteria in order to jointly estimate the external validity of the scale in a full structural model, thus extending the measurement model to a Multiple Causes Multiple Indicators (MIMIC) model. All models were estimated in the Mplus 6.1 software [20]. Model fit was assessed with the Comparative Fit Index (CFI), the Tucker Lewis Index (TLI) and the Root Mean Square Error of Approximation (RMSEA) following the recommendations of Yu on their interpretation (Evaluation of model fit indices for latent variable models with categorical and continuous outcomes. Unpublished dissertation, 2002; see Mplus website http://www.statmodel.com/download/Yudissertation.pdf).

For missing data, we employed the Full Information Maximum Likelihood (FIML) method which is naturally incorporated into structural equation models. In this full likelihood context model parameters and standard errors are estimated directly from the available data and the selection mechanism is ignorable under the Missing at Random (MAR) assumption [21, 22]. The basic goal of the FIML method of handling missing data is to identify the population parameter values that are most likely to have produced a particular sample of data and the discrepancy between the data and the estimated parameters is quantified by this likelihood. In this context the MAR assumption implies that all systematic selection effects depend on variables which are included in the models.

### Ethical approval

Ethical approval for the study was granted by Oxford A Research Ethics Committee. Governance approval was secured from all the participating NHS trusts.

## Results

### Measure development

The underlying conceptual framework for the model is described in detail elsewhere [5] and is summarized in Table 2. Of 31 criteria identified from the qualitative data, 18 were excluded based on our decision rules (see Table 2). The remaining 13 items were included in the draft psychometric measure (12 each for men and women). They related to psycho-physiological aspects of function (Q1-1 to Q1-8/9) and relational aspects of function (Q2 to Q5). Based on our data and the literature, we added eight further indicators that would allow respondents to self-rate their level of function: (Q6 to Q9 plus an item called ‘perception that no problem exists’ which was later excluded); plus three items that gave further information about the severity of any reported problems. These latter three items were excluded from the final measure based on the results of the psychometric analysis (see below). Table 2 shows the items included and excluded at the qualitative development stage.

**Table 2:** Items selected from qualitative development work

Items derived from qualitative development work	Question number in final measure (see “Appendix”)
*Psycho*-*physiological aspect*	
Desire for sex	Q1-1
Enjoyment	Q1-2
Lack of anxiety	Q1-3
Absence of discomfort/pain	Q1-4
Sexual arousal/excitement	Q1-5
Orgasm-ability to reach	Q1-6
Orgasm-not too early	Q1-7
Lubrication (F)/Erectile function (M)	Q1-8/9
*Severity if difficulty present*	
Duration since onset of difficulty	Item excluded based on psychometric analysis
Frequency with which symptoms occur	Item excluded based on psychometric analysis
Distress caused by Symptoms	Item excluded based on psychometric analysis
*Relational aspect*	
Balance in levels of desire	Q2
Compatibility in sexual preferences	Q3
Partner does not have sexual difficulties	Q4
Emotional connection	Q5
*Global self*-*rating aspect*	
Overall satisfaction	Q6
Overall lack of distress/worry	Q7
Not avoiding sex	Q8
Perception that no problem exists	Item excluded based on psychometric analysis
Not seeking professional help	Q9

Items excluded at the qualitative development stage
Decision to exclude based on the three decision rules (exclude if criterion overlaps with another; exclude if respondents view it as desirable rather than essential; exclude if associated with sexual function rather than part of construct itself)
*Functional sexual self*: Happy body feeling, able to give and receive pleasure, positive sexual identity, Confidence to communicate needs, positive motives to have sex
*Psycho*-*physiological*: Novelty, quality of orgasmic experience, actual frequency of sex, actual frequency relative to desired
*Relational*: Trust, warmth, feeling wanted, compatibility in motive for sex, compatibility in sexual roles/identities, reciprocity, chemistry
*Contextual*: Stress and tiredness, privacy

Cognitive pre-testing confirmed that the items derived from the criteria (see Table 1 and “Appendix”) were acceptable and understood as intended. Refinements were made to wording, filtering and response option formats, but no items were dropped. Average time to complete the measure was 6 minutes; participants considered it straightforward to complete (Flesch Reading Ease Score was 66.6, where acceptable range is between 60 and 70; this tool is available in MS word) and most participants did not require any assistance to use a laptop. The measure was felt to be relevant and acceptable by the two gay men in the sample (there were no lesbian women).

### Analysis and validation of final measure

We restricted analysis to participants who reported having sex in the past year. We began to examine the structure of the Natsal-SF with an Exploratory Factor Analysis (EFA) suitable for binary and ordinal variables. There were three eigenvalues larger than one, indicating that three latent factors were necessary to account for responses to the Natsal-SF items. At this stage we omitted several items that added no information to the model (see Table 2).

With the EFA results as a guide, we proceeded by testing restricted Confirmatory Factor Analysis (CFA) models for the Natsal-SF. We first estimated a model with three first order factors, following which we used a second order model where a higher order latent factor subsumes the three first order factors and a general specific model in which a global latent factor accounts for variation directly in all Natsal-SF items. According to the fit indices presented in Table 3, the General-Specific model had the best fit to the data. It was also equivalent between the general population and clinical samples, as well as between men with lower than desired and as much as desired sexual activity, as reflected in the good fit of an invariant between the groups general-specific model in which measurement parameters (thresholds, factor loadings and their associated standard errors) functioned equivalently in both groups. Additionally we established measurement invariance for gender and between different age groups. In other words, the measure functions equivalently across gender, age and clinical status.

**Table 3:** Criteria of model fit

	CFI	TLI	RMSEA
Unidimensional model	0.915	0.902	0.085
Second order model	0.914	0.898	0.098
General-Specific model	**0.963**	**0.951**	**0.064**
General-Specific model—Measurement Invariance^a^	0.961	0.957	0.047
General-Specific model—Measurement Invariance^b^	0.952	0.946	0.057

*CFI* comparative fit index, values >0.95 indicate good fit

*TLI* Tucker Lewis index, values >0.95 indicate good fit

*RMSEA* root mean square error of approximation, values <0.08 indicate good fit

^a^Invariant measurement model between general population and clinical samples

^b^Invariant measurement model between men with as much as desired and lower than desired sexual activity

In Table 4 we present the standardized factor loadings of all Natsal-SF items. Standardized factor loadings indicate the relative contribution of individual items to the overall score. The general standardized factor loadings capture the common variance between the 17 items, thus measuring problems in sexual functioning. The three specific factors (see Table 4) capture common variance between their allocated items, which is not due to problems with sexual functioning. All items loaded satisfactorily on the general Natsal-SF latent factor (0.493–0.912), with the exception of the “reached a climax more quickly than you would like” item that performed poorly but in the expected direction (r = 0.177, *p* < 0.05). This item remained in the model for theoretical reasons: premature ejaculation is known to be a common sexual function problem among men.^1^ Based on the selected general specific model, we estimated latent scores that reflect the Natsal-SF continuum. The estimated latent Natsal-SF scores were normally distributed, (Skewness = −0.116, Kurtosis −0.229) and ranged from −6.2 to 7.3, with high scores indicating the presence of sexual function problems.

**Table 4:** Standardised factor loadings derived from the General-Specific model

Q nmbr*	Measure items	Natsal-SF	SFS1	SFS2	SFS3
Q1-1	Lacked interest in having sex	0.657	0.301		
Q1-2	Lacked enjoyment in sex	0.678	0.566		
Q1-3	Felt anxious during sex	0.585	0.108		
Q1-4	Felt physical pain as a result of sex	0.605	0.188		
Q1-5	Felt no excitement or arousal during sex	0.618	0.639		
Q1-6	Did not reach/had trouble reaching a climax	0.493	0.499		
Q1-7	Reached a climax more quickly than you would like	0.177	−0.438		
Q1-8/9	Trouble getting or keeping an erection/uncomfortably dry vagina	0.534	0.059		
Q2	My partner and I share about the same level of interest in having sex	0.572		0.521	
Q3	My partner and I share the same sexual likes and dislikes	0.413		0.776	
Q4	My partner has experienced sexual difficulties in the last year	0.457		0.071	
Q5	How often would you say you feel emotionally close to your partner when you have sex together	0.472		0.361	
Q6	I feel satisfied with my sex life	**−**0.912			**−**0.402
Q7	I feel distressed or worried about my sex life	0.866			0.251
Q8	I have avoided sex because of sexual difficulties, either my own or those of my partner	0.813			0.459
Q9	Sought help or advice regarding sex life	**−**0.521			0.122

See “Appendix”

* Denotes question number in questionnaire

**Fig. 1:**
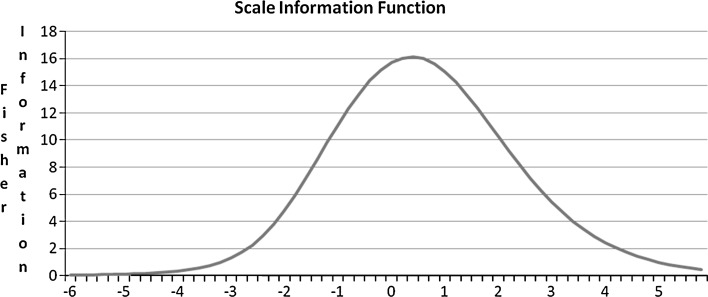
Natsal-SF scale information function

In Fig. 1 we present the Scale Information Function (SIF) of the general factor of the Natsal-SF. The SIF remained high across a wide range of values, with greatest score precision (maximum information/lowest standard error) being observed, as expected, towards mid range values (−1.6 to 2.4), which is desirable for a general population metric. This suggests that the effective measurement range of the Natsal-SF covers at least 55 % of the general population. In other words, the Natsal-SF measures very well in 55 % of the population and less well in the remaining 45 % (no scale is reliable for 100 % of the population).

The questionnaire was acceptable. In 13 items the missing data was <3 %. For the remaining 4 items the proportion missing was 35 %; these four items enquired about the sexual relationship and were only answered by those who had been in a sexual relationship for the year preceding the survey.

### External validity

At the second stage of our analysis we investigated the predictive power of the Natsal-SF general factor against well established external criteria. In Table 5 we present the estimated associations between the Natsal-SF and several external criteria. All estimated parameters were adjusted for each other, therefore providing conservative external validity tests for the Natsal-SF, compared to univariate analyses in which only the crude association between the Natsal-SF and each of the external criteria is tested. The Natsal-SF general factor had a significant positive association with being a clinical respondent (OR = 2.667, *p* < 0.001), a negative association with having sex as frequently as desired (OR = 0.637, *p* < 0.001, men only), a positive association with the FSFI-6 (B = 0.572, *p* < 0.001), as well as the BSFQ (B = 0.705, *p* < 0.001). Furthermore we observed a positive association between the Natsal-SF general factor and reporting “fair” or “bad” health (OR = 1.171, *p* < 0.05), as well as with “feeling depressed or hopeless nearly every day” (OR = 1.202, *p* < 0.001). We also observed a negative association between the natsal-SF and being “satisfied with life at the moment” (OR = 0.837, *p* < 0.001). On the contrary, we did not observe a significant association between the Natsal-SF general factor and weekly alcohol use (OR = 1.061, *p* > 0.05). We note that all significant associations between the Natsal-SF general factor and the external criteria were in the expected direction, confirming the external validity of the Natsal-SF.

**Table 5:** Regression estimated parameters of the association between the Natsal-SF general factor and external criteria

	Clinical	FSFI-6	BSFQ	Self rated health	Depression	Well-being	Alcohol
Natsal-SF	2.667**	0.572**	0.705**	1.171*	1.202*	0.839*	1.061
Gender	0.231**			1.106	1.275	0.834	0.514**
Age	0.963*	0.126**	−0.001*	**1.043**	0.994	1.015	1.031**
Ethnicity	0.391**	0.056	**−**0.031	1.141	1.152	0.994	2.144*
Marital status	**1.979** ^*^	**−**0.085	−0.070*	1.119	0.961	2.611**	1.099
Social grade		**−**0.011	0.025	**0.601**	0.715	0.785	1.266
Working Status		**−**0.009	**−**0.034	1.310	0.795	1.117	0.951
Self rated health	0.380*	0.098*	0.010		1.597*	0.274**	0.849
Depression	0.700	−0.135*	**−**0.057	**1.617**		0.103**	1.404
Well-being	0.684	**−**0.084	**−0.123**	**0.272**	0.105**		1.193
Alcohol	1.348	0.071*	0.048	0.849	1.388	1.153	

* With the exception of the linear regression on FSFI (where the standardized coefficient is reported), all other models are multiple logistic regressions and Odds Ratios are reported

** Highlighted parameters are significant, ** p < 0.001, * p < 0.05

*** The first model (“clinical”) was estimated on the combined general population and clinical respondents sample. The model including the FSFI was estimated on general population sampled women, whereas the model including the BSFQ was estimated on general population sampled men. All other models were estimated on the pooled general population sample

## Test–retest reliability

The test–retest reliability of the Natsal-SF general factor was r = 0.72, *p* < 0.001, in a sample of 144 participants who responded to the 17 Natsal-SF items at follow up two weeks after completing the first survey.

## Discussion and conclusion

We found the Natsal-SF to be reliable, valid and able to discriminate between clinical and general population groups. It provides a measure of sexual function in the last year. By including items on distress and relationships, and by being relevant to all regardless of sexual lifestyle, it addresses some of the key gaps in current measurement design.

For implementers of large-scale epidemiological surveys there are several advantages to this measure. It is brief and the questions are non-intrusive and easy to understand. Programme filtering means that respondents only see questions relevant to their experience.

As previously outlined, this is a measure of sexual function, which according to our development work is about the absence of sexual function problems, a positive sexual relationship, feeling sexually satisfied and an absence of personally felt distress. It is important to note that the items on individual sexual function problems do not equate to a clinical diagnosis of specific dysfunction. Our development work [5] suggested that the construct of sexual function is as much relational and psychological as it is biomedical, and so the avoidance of a clinical diagnosis may be seen as an advantage. A limitation of the methodology of this study has been the use of an internet panel as a proxy to the general population [23–25]. However the forthcoming Natsal-3 survey will provide opportunity to further validate the measure on a community based random probability sample. The poor performance of the item “reached a climax more quickly than you would like” is puzzling. One possible explanation is that some respondents tick ‘yes’ to this item, not because they feel they have a problem with premature ejaculation, but simply because they see delaying climax further as an ideal; they actually rate their function as fine. Our data supports this hypothesis: less than a third (29 %) of general population respondents reported feeling fairly or very distressed about this experience. On average, for all the other problems, 53 % reported feeling fairly or very distressed.

The inclusion of items measuring persistence of symptoms, severity of symptoms and associated distress has been shown to impact on prevalence estimates [9, 26]. Our measure originally included these items but they were excluded from the final measure because they did not add further information. Similarly we asked respondents who reported avoiding sex, why they had done so, and this item was excluded for the same reason. However, survey implementers may wish to include these items in their questionnaire because they add useful detail when examining sexual function difficulties separately.

The possibilities for future use of this measure are exciting. It is our hope that it will be widely used in community surveys. Being brief, it is also likely to be attractive to clinicians as a clinical screening tool, although a separate study would be required to assess its validity in this context.

## Appendix:

**The Natsal-SF: A measure of sexual function for community surveys**

ASK ONLY IF RESPONDENT HAS HAD SEX IN PAST YEAR (ALL OTHER RESPONDENTS SHOULD BE ROUTED TO Q6)

DISPLAY TO RESPONDENTS

The next few questions are about your sex life. Some questions use the term ‘having sex’. By this we mean vaginal, oral, or anal sexual intercourse

Some people go through times when they are not interested in sex or find it difficult to enjoy sexual activities. The questions that follow are about some common difficulties that people experience.

Q1

In the last year, have you experienced any of the following **for a period of 3 months or longer?**

Please type in the number of every one that you have experienced **for a period of 3 months or longer**.

You can type in more than one number by pressing the spacebar between each number.

If you have not experienced any please type in ‘10’.

[Multiple responses allowed, except for code 10]

1. Lacked interest in having sex
2. Lacked enjoyment in sex
3. Felt anxious during sex
4. Felt physical pain as a result of sex
5. Felt no excitement or arousal during sex
6. Did not reach a climax (experience an orgasm) or took a long time to reach a climax despite feeling excited/aroused
7. Reached climax (experienced an orgasm) more quickly than you would like
8. Had an uncomfortably dry vagina (asked of women only)
9. Had trouble getting or keeping an erection (asked of men only)
10. I did not experience any of these

ASK IF RESPONDENT HAS HAD SEX IN PAST YEAR **AND** HAS BEEN MARRIED OR IN CIVIL PARTNERSHIP OR LIVING WITH A PARTNER AS A COUPLE FOR AT LEAST ONE YEAR (ALL OTHER RESPONDENTS SHOULD BE ROUTED TO Q6)

You previously mentioned that you have been (insert relevant status e.g. “in a civil partnership”) for at least one year. Thinking about your relationship with this partner in the last year, how much do you agree or disagree with the following statements.

Q2

“My partner and I share about the same level of interest in having sex”

1. Agree strongly
2. Agree
3. Neither agree nor disagree
4. Disagree
5. Disagree strongly

Q3

“My partner and I share the same sexual likes and dislikes”

1. Agree strongly
2. Agree
3. Neither agree nor disagree
4. Disagree
5. Disagree strongly

Q4

“My partner has experienced sexual difficulties in the last year”

1. Agree strongly
2. Agree
3. Neither agree nor disagree
4. Disagree
5. Disagree strongly

Q5

“I feel emotionally close to my partner when we have sex together”

1. Always
2. Most of the time
3. Sometimes
4. Not very often
5. Hardly ever

ASK ALL

The next few questions ask about your sex life in the last year. An individual’s sex life includes their sexual thoughts, sexual feelings, sexual activity and sexual relationship.

Thinking about your sex life in the last year, how much do you agree or disagree with the following statements:

Q6

“I feel satisfied with my sex life”

1. Agree strongly
2. Agree
3. Neither agree nor disagree
4. Disagree
5. Disagree strongly

Q7

**“**I feel distressed or worried about my sex life.”

1. Agree strongly
2. Agree
3. Neither agree nor disagree
4. Disagree
5. Disagree strongly

Q8

**“**I have avoided sex because of sexual difficulties, either my own or those of my partner”

1. Agree strongly
2. Agree
3. Neither agree nor disagree
4. Disagree
5. Disagree strongly

Q9

Have you sought help or advice regarding your sex life from any of the following sources in the last year?

You can type in more than one number by pressing the spacebar between each number.

If you have not sought any help or advice, type in ‘11’.

[Multiple responses allowed, except code 11]

1. Family member/friend
2. Information and support sites on the internet
3. Self-help books/Information leaflets
4. Self-help groups
5. Helpline
6. GP/Family doctor
7. Sexual health/GUM/STI clinic
8. Psychiatrist or psychologist
9. Relationship counsellor
10. Other type of clinic or doctor
11. Have not sought any help

**END**

## Footnotes

1 We estimated the general-specific model excluding this item and obtained the following indices of fit: CFI = 0.957, TLI = 0.941, RMSEA 0.069. They all indicate the acceptable fit of the model, although their values are slightly inferior for those obtained from the original model.
